# NeRF-based 3D reconstruction pipeline for acquisition and analysis of tomato crop morphology

**DOI:** 10.3389/fpls.2024.1439086

**Published:** 2024-10-24

**Authors:** Hong-Beom Choi, Jae-Kun Park, Soo Hyun Park, Taek Sung Lee

**Affiliations:** Smart Farm Research Center, Korea Institute of Science and Technology (KIST), Gangneung, Gangwon, Republic of Korea

**Keywords:** 3D phenotyping, neural radiance fields, automated growth measurement, point cloud, greenhouse application

## Abstract

Recent advancements in digital phenotypic analysis have revolutionized the morphological analysis of crops, offering new insights into genetic trait expressions. This manuscript presents a novel 3D phenotyping pipeline utilizing the cutting-edge Neural Radiance Fields (NeRF) technology, aimed at overcoming the limitations of traditional 2D imaging methods. Our approach incorporates automated RGB image acquisition through unmanned greenhouse robots, coupled with NeRF technology for dense Point Cloud generation. This facilitates non-destructive, accurate measurements of crop parameters such as node length, leaf area, and fruit volume. Our results, derived from applying this methodology to tomato crops in greenhouse conditions, demonstrate a high correlation with traditional human growth surveys. The manuscript highlights the system’s ability to achieve detailed morphological analysis from limited viewpoint of camera, proving its suitability and practicality for greenhouse environments. The results displayed an R-squared value of 0.973 and a Mean Absolute Percentage Error (MAPE) of 0.089 for inter-node length measurements, while segmented leaf point cloud and reconstructed meshes showed an R-squared value of 0.953 and a MAPE of 0.090 for leaf area measurements. Additionally, segmented tomato fruit analysis yielded an R-squared value of 0.96 and a MAPE of 0.135 for fruit volume measurements. These metrics underscore the precision and reliability of our 3D phenotyping pipeline, making it a highly promising tool for modern agriculture.

## 1 Introduction

Digital phenotypic analysis is increasingly recognized as an essential element in the accurate morphological analysis of crops and is becoming increasingly important in various application areas (Tripodi et al., 2022). It goes beyond simple observations to digitalize and quantify the crop’s genetic trait expressions. In addition, phenotypic analysis is complexly linked to environmental data, promoting an informed decision-making process to optimize cultivation conditions and improve crop yields.

Two-dimensional (2D) imaging is primarily used in computer vision-based phenotyping studies. For example, segmentation algorithms analyze the number of pixels within a segment to calculate the projected area for area analysis (Fonteijn et al., 2021). Methods, such as the convex hull method, have also been employed to analyze the growth state of crops (Du et al., 2021). Moreover, extracted silhouettes can regressively estimate the crop volume (Concha-Meyer et al., 2018). Despite their usefulness, these methods fail to capture the complete complexity of plant morphology. When reducing 3D structures to 2D representations, significant data that aid the comprehensive understanding of plant health and development, such as leaf curvature, area, and overall plant volume, can be lost.

Several methods using RGB-D cameras and key-point detection (Vit et al., 2019) have been proposed to obtain 3D information about crop morphology. For example, structure from motion-based methods extract phenotyping elements of greenhouse crops from RGB photos captured from multiple angles (Li et al., 2020; Wang et al., 2022). Alternatively, laser scanners can obtain more precise 3D plant models (Schunck et al., 2021). Additionally, obtaining the 3D form of crops is essential for future agriculture, as it enables phenotyping and advanced applications, such as light interception analysis (Kang et al., 2019). However, 3D phenotyping in greenhouse environments poses several challenges. First, even with the light-diffusing film in greenhouses, scattered sunlight can still create substantial noise during measurements using active 3D imaging approaches (such as consumer-level depth cameras like Intel RealSense L515, D435, or laser scanners) (Neupane et al., 2021; Maeda et al., 2022; Harandi et al., 2023). Second, focusing on high-interest positions in greenhouse crops, such as tomatoes, results in low productivity for large and heavy measurement equipment. Third, narrow spacing, typical of greenhouses, makes obtaining sensor data from various angles difficult for 3D model acquisition.

The emerging neural radiance field (NeRF) technology (Gao et al., 2022) offers a new direction for 3D phenotyping. NeRF uses a fully connected neural network to model volumetric scene features and render images from various viewpoints, capturing the 3D structure of a scene. NeRF is robust and can represent complex morphological structures with fewer and more sparsely distributed input images, making it suitable for 3D phenotyping. The time incurred in training NeRFs, which was previously tens of hours, has significantly improved to just minutes with the advent of Instant-NGP, applying hash-encoding-based positional encoding (Müller et al., 2022). Moreover, the user-friendly Nerfstudio framework has made the application and training of NeRF more accessible (Tancik et al., 2023). In agriculture, many applications are underway, including applying semantic segmentation techniques to assist robots’ scene understanding in greenhouse (Smitt et al., 2024) or analyzing crops with complex structures (Saeed et al., 2023).

The proposed pipeline encompasses automated RGB image acquisition through a specialized greenhouse robot platform with a 6-degrees of freedom (6-DoF) robot arm. It also includes acquiring dense point-cloud data utilizing NeRF technology, followed by extracting detailed morphological information from the data. A key aspect of this pipeline is adopting a forward-facing capture technique by operating from a fixed position with a limited field of view, which means that the crops are not captured from a full 360-degree angles at ground level. This limitation aligns more realistically with the practical constraints of greenhouse environments. Despite this limitation, the approach provides the noninvasive measurement accuracy of critical crop parameters, such as length, leaf area, and fruit volume. The application of this method was demonstrated through nondestructive measurements of tomato crops in conditions mirroring actual greenhouse environments. The morphological data obtained were then compared with that acquired through traditional human growth surveys, allowing for a thorough evaluation of the measurement accuracy. The main contributions of this study are as follows:

- A system and data processing pipeline were presented to obtain 3D crop models in greenhouse environments based on images automatically collected by unmanned robots.
- The proposed pipeline demonstrates obtaining decent point-cloud data of crop images from limited viewpoints, showcasing a realistic method for greenhouse environments.
- The proposed 3D plant model was used to measure the following: 1D information, such as stem thickness, node length, and flower position height; 2D information, such as leaf area; 3D information, such as fruit volume. These measurements were compared with actual measurements to demonstrate the suitability of the proposed pipeline for use in growth surveys.

## 2 Materials and methods

### 2.1 Pipeline overview

**Figure 1:**
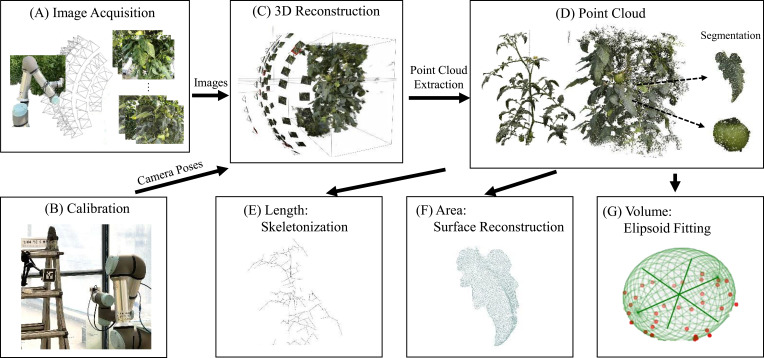
3D phenotyping pipeline for tomato crop analysis. The steps are as follows: **(A)** Image acquisition using a 6-DoF robot, **(B)** Camera calibration, **(C)** NeRF training for 3D reconstruction, **(D)** Point cloud extraction and segmentation, **(E)** Skeletonization for inter-node length measurement, **(F)** Surface reconstruction for leaf area measurement, and **(G)** Ellipsoid fitting for fruit volume estimation.

A 3D phenotyping pipeline was presented; 3D point clouds based on RGB images obtained from multiple viewpoints were reconstructed using a robot for nondestructive constraint-overcoming measurements in greenhouse environments. The proposed pipeline comprises seven elements, as shown in **Figure 1**: (A) Acquiring images using a 6-DoF robot from various viewpoints. (B) Acquiring camera poses from images and calibrating these poses to a meter scale. (C) Training NeRF based on the acquired images and camera poses. (D) Extracting and segmenting the point cloud based on the color and depth rendering results from the NeRF. (E) Skeletonizing to identify connections between plant organs and to extract length information. (F) Reconstructing the surface on the segmented leaf part of the point cloud and calculating the area from the obtained surface. (G) Fitting an ellipsoid to the segmented fruit part of the point cloud and estimating the fruit volume by calculating the ellipsoid volume.

### 2.2 Image acquisition

**Figure 2:**
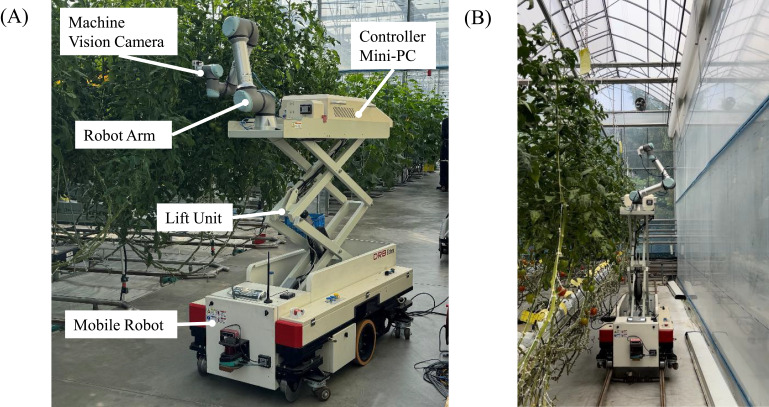
**(A)** Configuration of the greenhouse unmanned robot platform. **(B)** Example of a robot taking measurements in a greenhouse.

The robot illustrated in **Figure 2A** was developed in a previous study (Cho et al., 2023) and was employed to facilitate image acquisition in the greenhouse. The base comprises a smart-farm robot platform that controls mobility and provides power. A 6DoF manipulator, UR-5e, with a maximum reach of 850 mm, is mounted on top of this platform. A machine-vision camera, an IDS U3-36L0XC, is attached to the end effector of this robot arm and designed for photographic capture. This camera has a 4200 x 3120-pixel resolution and a frame rate of 20 frames per second. It is connected to a mini-PC via USB to control the image-capturing process. This mini-PC is connected to the robot arm through a LAN and is equipped with integrated software to control the image-capturing process and robot arm.

In addition, the robot arm-based image acquisition system includes a lift unit for transporting along the Z-axis. This allows maneuvering the robot arm to the desired crop and then vertically to the area of interest using the lift. In the greenhouse environment where the validation was conducted, the average distance between crops was 40 cm, and the distance between lanes was 150 cm. As shown in **Figure 2B**, the robot was deployed in the field to capture images. The image acquisition process, depicted in **Figure 1A**, involves capturing images from 64 different poses. These poses are arranged on the surface of a virtual sphere with a radius of 60 cm (the average distance between the crop and the robot arm), centered on the target area of interest. The dome is formed by the portion of the sphere that falls within the robot arm’s reach, creating a set of poses that cover the necessary angles.

### 2.3 NeRF-based 3D reconstruction

NeRF presents a novel approach to 3D scene reconstruction by synthesizing photorealistic images using deep learning. A fully connected neural network is used to model volumetric scene features, rendering complex 3D scenes from 2D image sets. NeRF can interpolate and extrapolate new views from sparse input data, creating highly detailed and coherent 3D reconstructions. The underlying mechanism involves learning the color and density distribution of light in a scene as a function of the position and viewing direction. In NeRF, each pixel is represented as a ray. For each ray, its position information (x, y, z), representing the 3D coordinates in space, and direction (θ, φ), representing the viewing angles, are fed into a multilayer perceptron (MLP). The MLP outputs the RGB value and transparency (σ) for that ray. This process essentially captures the light and color information passing through the scene and the density distribution along the rays, providing the basis for reconstructing a 3D model from 2D images. In other words, the NeRF model can receive a 5-dimensional vector, including position and viewing direction, as input and provide RGB values and depth images as output.

Preprocessing to acquire the pose information of the images during the image-acquisition phase is essential for the NeRF to learn and reconstruct a scene. The pose information (x, y, z, θ, and φ) is obtained using structure from motion software called COLMAP (Schonberger and Frahm, 2016). In our pipeline, the UR-5e robot arm, which supports pose repeatability within 0.03 mm, requires running the COLMAP process only once for each set of pre-defined robot arm’s poses. The calibration process is shown in **Figure 1B**.

A marker with known physical measurements were used during calibration. Specifically, a 30 cm by 30 cm printed marker was placed 75 cm away from the robot arm’s base to replicate crop measurement conditions. By capturing a single scene with this setup, we were able to obtain the COLMAP results, which provided the marker’s coordinates in the reconstructed scene. These coordinates were then matched with the actual known dimensions of the marker, allowing us to determine the scale factor that converts the displacement output (x, y, z) from COLMAP into a metric scale, but also enabled us to reuse the calibrated camera poses in subsequent image captures. As a result, there is no need to recalculate the poses using COLMAP each time, simplifying the NeRF input process.

The Nerfacto model within the NerfStudio framework (Tancik et al., 2023), chosen for its combination of various NeRF-related research advantages, aligns well with the proposed 3D phenotyping pipeline. Despite the excellent pose repeatability of the robot arm, Nerfacto’s camera pose refinement (Wang et al., 2021) capability is crucial for minimizing potential noise in the results. In addition, hash encoding (Müller et al., 2022) significantly enhances learning speed, which enhances the overall efficiency of the pipeline. The proposal sampler (Barron et al., 2022) in Nerfacto, which focuses sample locations on the regions that contribute the most to the final rendering, particularly the first surface intersection, is essential for capturing complex crop details. This focused sampling approach is integral to accurately depicting the intricate morphological traits for detailed phenotypic analysis.

During training, we employed the Nerfacto model in Nerfstudio version 0.3.4, utilizing the default training parameters. However, because we were solely focused on point-cloud acquisition, we did not partition the validation set, and instead, adjusted the number of iterations to 20,000. The training was conducted on a workstation (AMD Ryzen™ Threadripper™ PRO 5975WX, 256GB RAM, NVIDIA RTX4090), and completed in approximately 5 minutes. After training, NeRF’s RGB render output and depth map output can be mapped for all camera poses included in the training set and sampled as a point cloud. This process utilized the implementation built into the NeRFStudio framework.

### 2.4 Phenotypic trait extraction

In this study, we extract phenotypic traits from point clouds generated by an earlier pipeline, focusing on inter-node length, leaf area, and fruit volume. We applied Laplacian-based contraction (LBC) (Cao et al., 2010) to the point cloud to extract length information, leading to skeletonization. Skeletonization reduces the point cloud to a more manageable representation and emphasizes the structural aspects of the plants. Because the skeleton resulting from the LBC is a collection of discontinuous points, we applied the minimum spanning tree (MST) algorithm (Meyer et al., 2023) to create a more coherent structure. The MST algorithm transforms the disconnected points into a graph-like structure, effectively representing the plant’s physical structure. Thus, the nodes in the skeleton can be aligned with the actual nodes of the crop stem, accurately representing the plant morphology. The final topology graph, extracted from the point cloud, has nodes whose coordinate system corresponds to the original point cloud. Consequently, the Euclidean distance between two points of interest in this graph represents the distance between the crop nodes.

**Figure 3:**
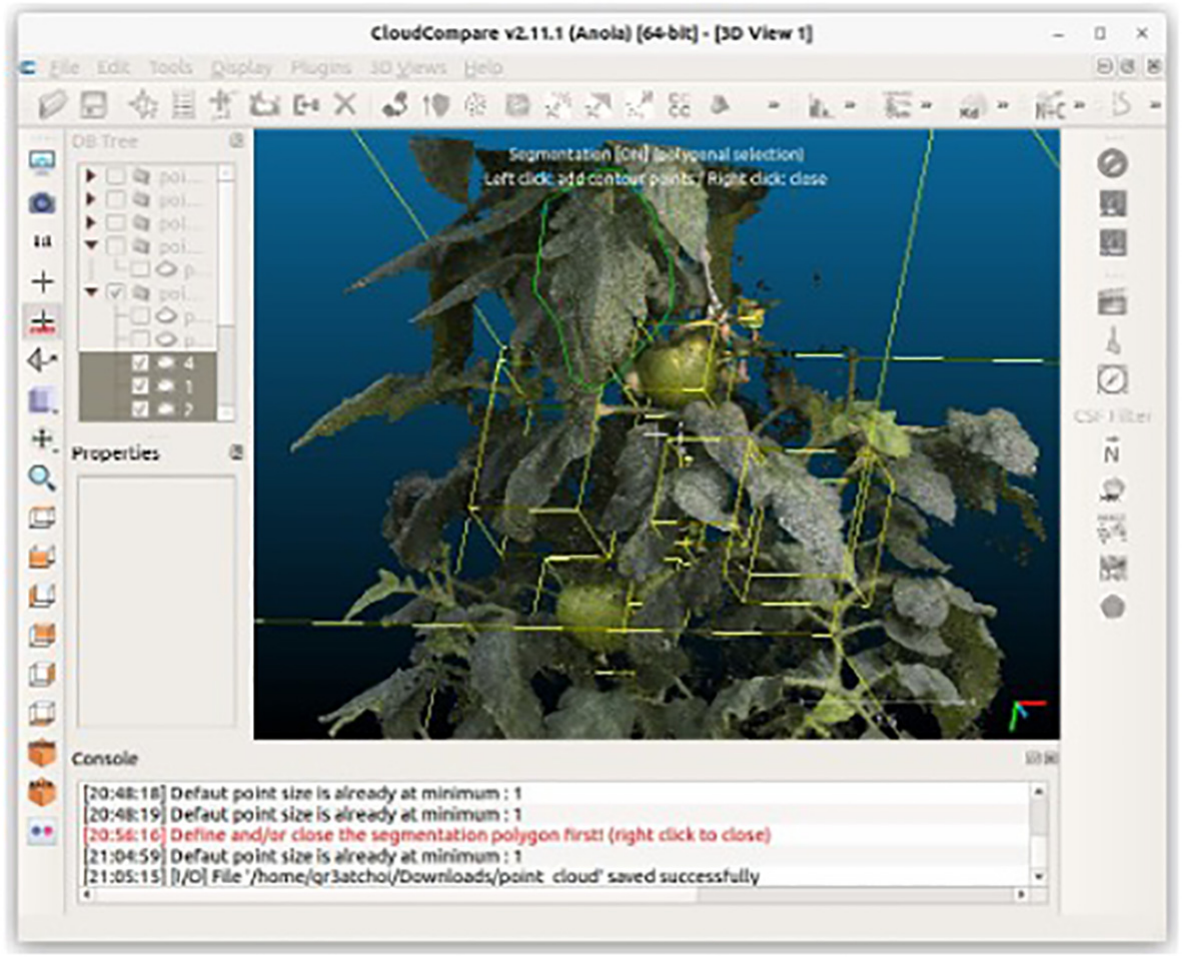
Manual segmentation of leaves and fruits using CloudCompare. Segmentation is performed by drawing a polygon (green lines) using the clipping tool. Afterward, a point cloud segmented with a yellow cuboid is displayed.

Extracting leaf area and fruit volume measurements requires preprocessing involving point-cloud segmentation. In this pipeline, we manually carried out this segmentation using CloudCompare (2023), as illustrated in **Figure 3**. Manual segmentation in CloudCompare allows the precise separation of different components of the point cloud, specifically distinguishing leaves, and fruits from other parts of the plant.

To calculate the leaf area, we first performed surface reconstruction on the segmented point cloud of the leaves. The total area was calculated as the sum of the triangular areas forming the mesh obtained from this reconstruction. However, accurately reconstructing a typically thin leaf structure requires noise removal near the leaf surface. Our pipeline incorporates a moving least squares (MLS) technique to address this (Alexa et al., 2001). MLS effectively converges points near the leaf surface while preserving the natural curvature and shape of the leaves (Boukhana et al., 2022). Next, the ball pivoting algorithm (BPA) was employed to generate the final mesh of the leaf. BPA works by rolling a ball of a specified radius over points to create a mesh, adeptly bridging gaps between points while maintaining the integrity of the leaf’s shape.

Finally, we employed ellipsoid fitting to estimate the volume of the segmented tomato fruits. Our pipeline uses input images captured from limited angles rather than from a full 360-degree view, which inevitably limits the measurement of the rear part of the tomato. However, despite this limitation, the tomato volume can be approximated by fitting an ellipsoid to the point cloud representing the measured portion of the tomato.

Ellipsoid fitting in this context is a simple but practical approach for volume estimation when complete data coverage is not feasible (Sari and Gofuku, 2023). By modeling the visible part of the tomato as an ellipsoid, we extrapolate the unmeasured portion, assuming symmetry and typical shape characteristics of tomatoes.

Fitting minimizes the size of the squared distance from the points to the ellipsoid surface, leading to the estimation of semi-axes *a*, *b*, and *c*. The optimization can be represented by minimizing the following function:


(1) $$\begin{array}{c} \;f\left(a,b,c\right)=\sum_{i=1}^{M}{\left(\frac{x_{i}^{2}}{a^{2}}+\frac{y_{i}^{2}}{b^{2}}+\frac{z_{i}^{2}}{c^{2}}-1\right)}^{2} \end{array}$$

where $\left(x_{i},\;y_{i},\;z_{i}\right)$ are the coordinates of the *i*-th point in the point cloud, and M is the total number of points in the point cloud. Optimization was performed using the least-squares method. Through the optimization, volume *V* of the ellipsoid fitted to the tomato point cloud can be obtained.

### 2.5 Ground truth measurement

To evaluate the accuracy of the proposed pipeline, we describe the ground-truth measurement methods conducted alongside image capturing. The results obtained by skilled cultivators using tape measures were used as the ground truth for measuring the node length. However, considering the node extraction based on skeletonization in our study, measurements were centered on the point where the branches diverged as much as possible.

**Figure 4:**
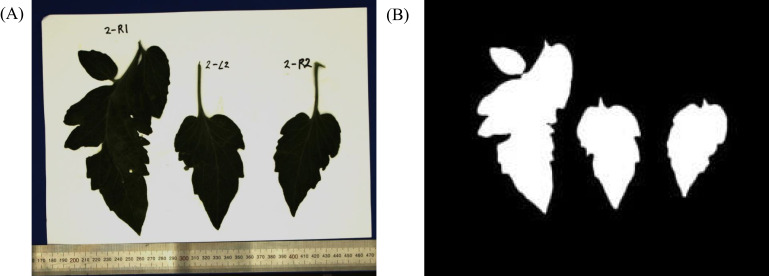
Obtained color-image **(A)** and background removal **(B)** for leaf area calculation.

For the leaf area, leaves were cut, affixed to paper, and photographed in a controlled studio environment, with the camera positioned directly above at a distance of 40 cm, ensuring a perpendicular angle. An example of a photographed leaf is shown in **Figure 4A**. The ruler is included to facilitate the conversion between pixel units and metric scale. Subsequently, binary processing was applied to these images to create silhouettes of the leaves, as shown in **Figure 4B**. The leaf area was then determined by calculating the pixel area of the silhouette in square centimeters ( $cm^{2}$ ) using a scale factor obtained from 1 cm pixels on the ruler.

Finally, for volume measurement, we utilized the principle of buoyancy, which calculates the volume of an object based on the weight and force required to submerge it in water (Concha-Meyer et al., 2018). The weight [g] was measured using a scale with a resolution of 0.05 g. A glass beaker filled with water was placed on the scale, and its tare function was used to adjust the reading to zero. The fruit, attached to a wire, was quickly submerged in water and positioned at the center of the beaker. The reading, which reflects the weight of the submerged fruit and the weight when pressed down by the wire, was recorded and represents the volume of the fruit [ $\text{c}m^{3}$ ].

## 3 Results

For validation, we measured the growth points of 16 tomato crops at the upper parts and the fruit clusters of 16 tomato crops at the lower parts, resulting in 32 image sets, each comprising 64 multi-view images. From these, we measured a total of 47 inter-node lengths, including 1 inter-node length above and 1-2 inter-node lengths below the topmost flower cluster for each plant. In each of the lower part image sets, we measured 2-3 leaves and 1-2 fruits. These selections were based on factors such as size, shape, and proximity to the robot arm to ensure diversity, resulting in measurements of 37 leaf areas and 20 fruit volumes in total. All measurements were conducted concurrently with ground-truth measurement.

**Figure 5:**
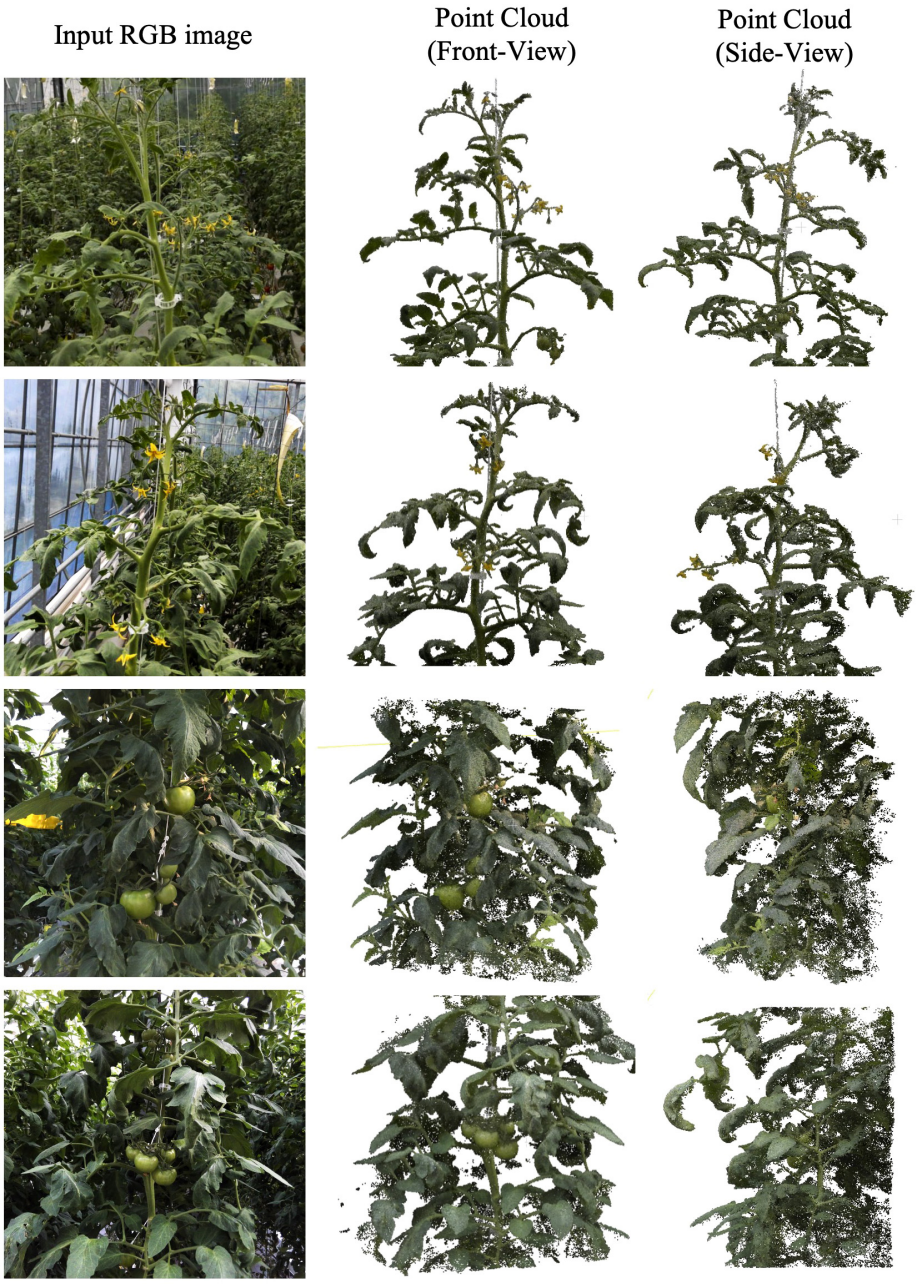
Front and side view appearance of point-cloud created from the input RGB images. The top and bottom two rows represent points near the growing point and fruit cluster, respectively.

**Figure 5** shows the extracted point clouds, illustrating two growth points and two fruit clusters. The front-view representation displays a dense formation of the point cloud, as captured from the angle at which the images were captured. However, the side view, representing angles not captured during imaging, shows reduced performance, especially in regions not directly imaged.

**Figure 6:**
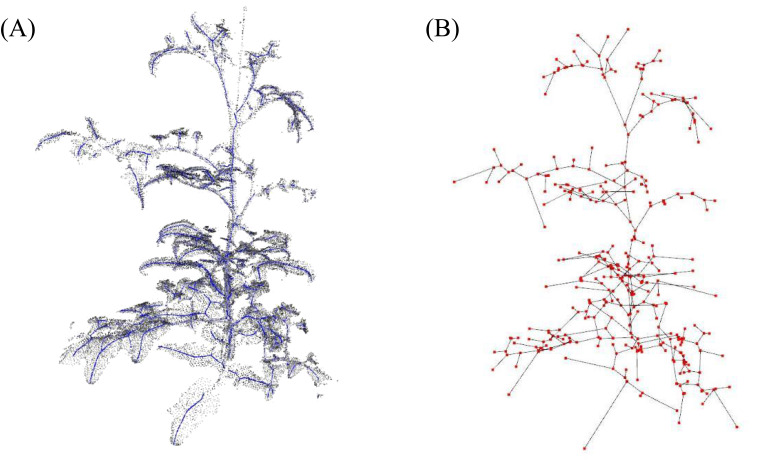
**(A)** Skeletonization process through applying Laplacian-based contraction to point cloud and **(B)** applying minimum spanning tree algorithm to the skeleton; blue and red dots indicate skeleton and nodes, respectively.

**Figure 6** presents the skeletonization results performed to measure the node-to-node lengths. In **Figure 6A**, the skeleton created through LBC is overlaid on the original point-cloud data as blue dots. **Figure 6B** shows the application of the MST algorithm to this skeleton; the red dots represent nodes, and the connections between them are depicted.

**Figure 7:**
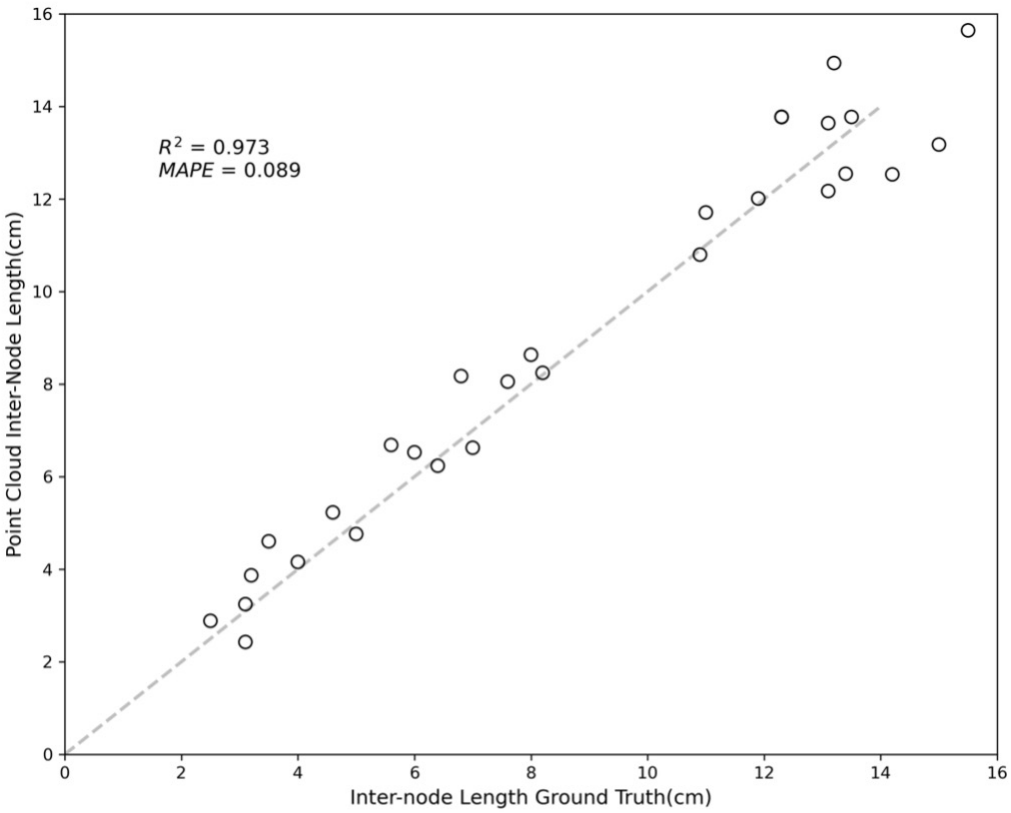
Comparison between inter-node length from NeRF and ground truth.

A comparison between the distances measured among the red dots and the node-to-node lengths measured manually is shown in **Figure 7**. The results showed an R-squared value of 0.973 and a mean absolute percentage error (MAPE) of 0.089, indicating a accuracy in the skeletonization and subsequent measurements. The error sources can be attributed to fundamental differences in the measurement approaches; the point cloud data measure lengths based on the central coordinates of the plant nodes, while the tape measure records lengths over the plant’s surface. The discrepancy between the two methods may account for the minor measurement variations. Despite these differences, the close correlation demonstrates the effectiveness of the skeletonization process in accurately capturing the crop’s physical dimensions.

**Figure 8:**
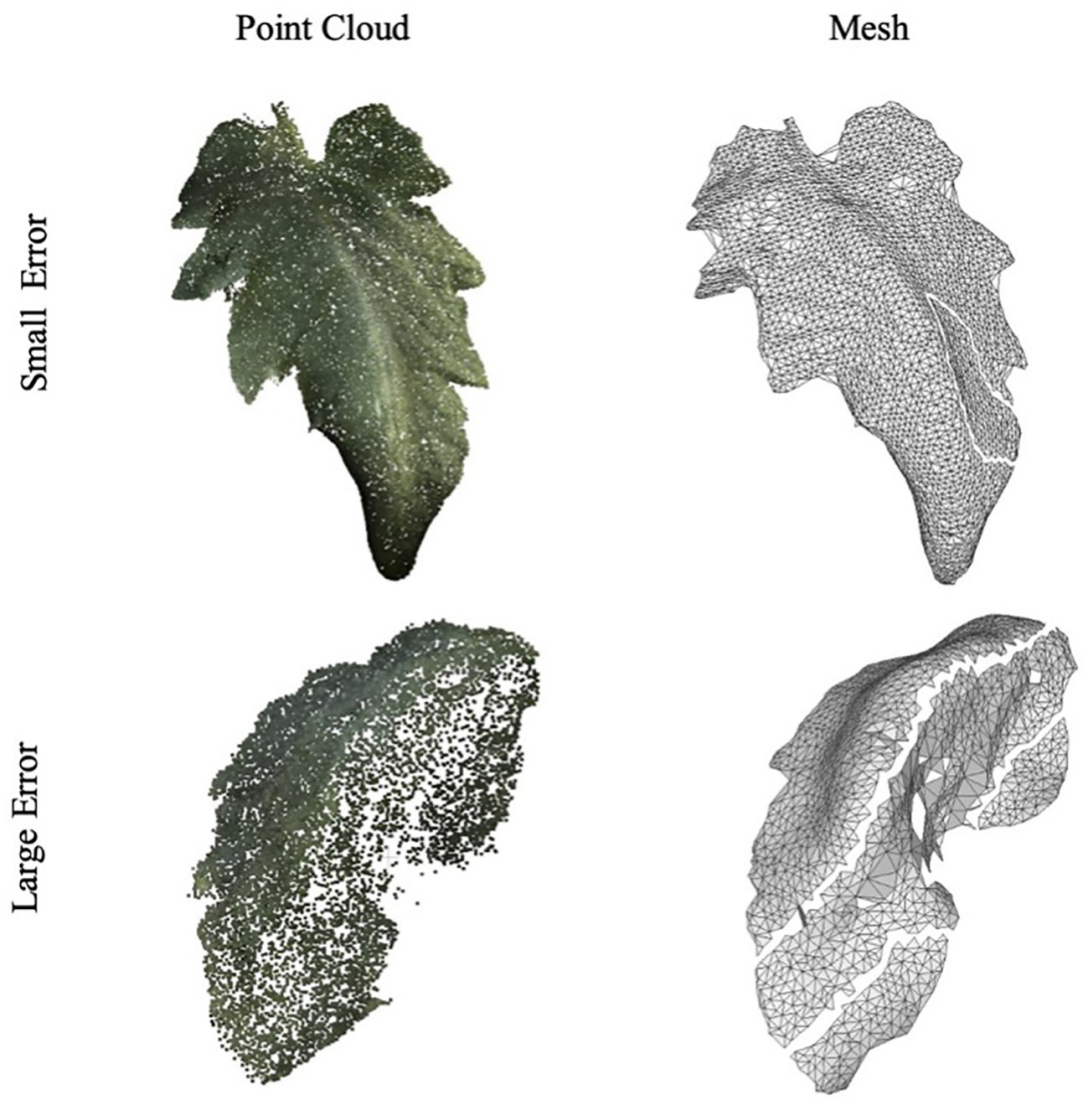
Example of leaf point cloud and surface reconstruction. A good example with a small error is at the top, and a bad example is at the bottom.

**Figure 8** showcases examples of the segmented leaf point cloud and the meshes reconstructed using MLS and BPA. The top two samples demonstrate instances with minimal error, serving as representative examples of high accuracy, while the bottom two samples exhibit significant discrepancies, highlighting cases with large errors.

**Figure 9:**
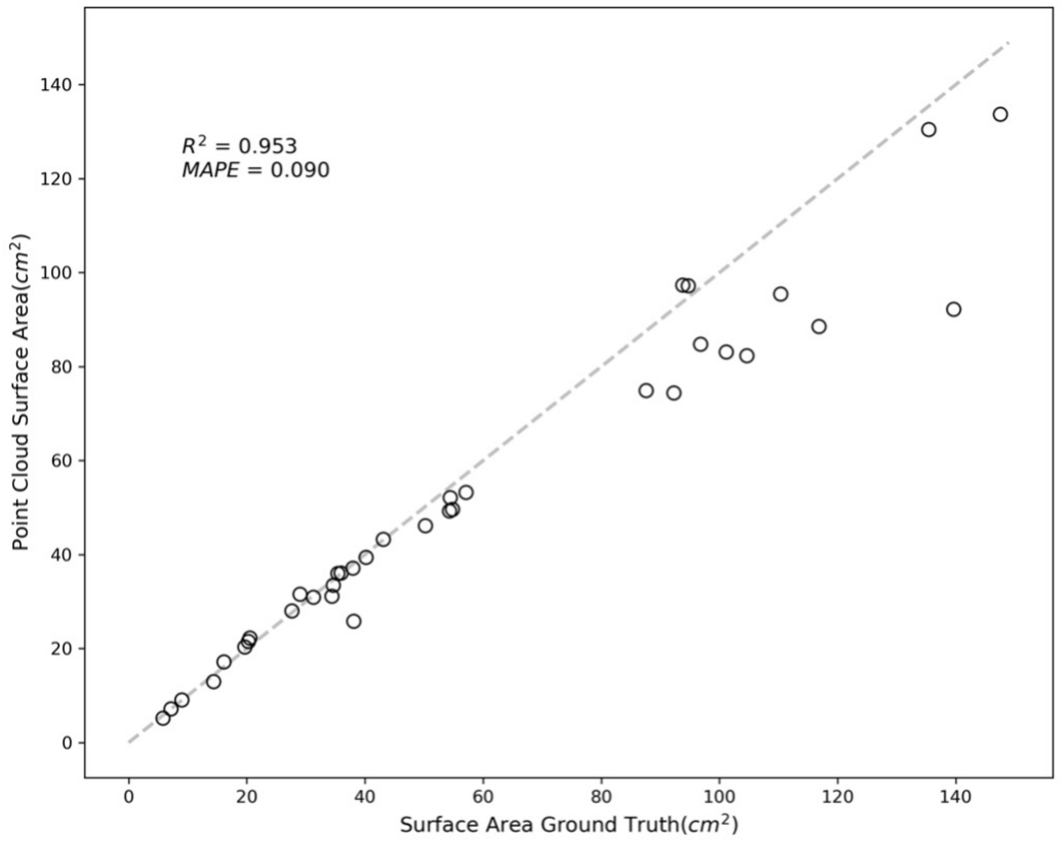
Comparison of surface area from NeRF and ground truth.

The variance in accuracy between these two sets of samples is attributed to the image-capturing angle. Samples with greater errors include leaves that were curled or rolled up, resulting in one side not being adequately captured. This lack of complete data led to inaccuracies in the reconstruction process. **Figure 9** further illustrates this point, with an R-squared value of 0.953 and a MAPE of 0.090.

**Figure 10:**
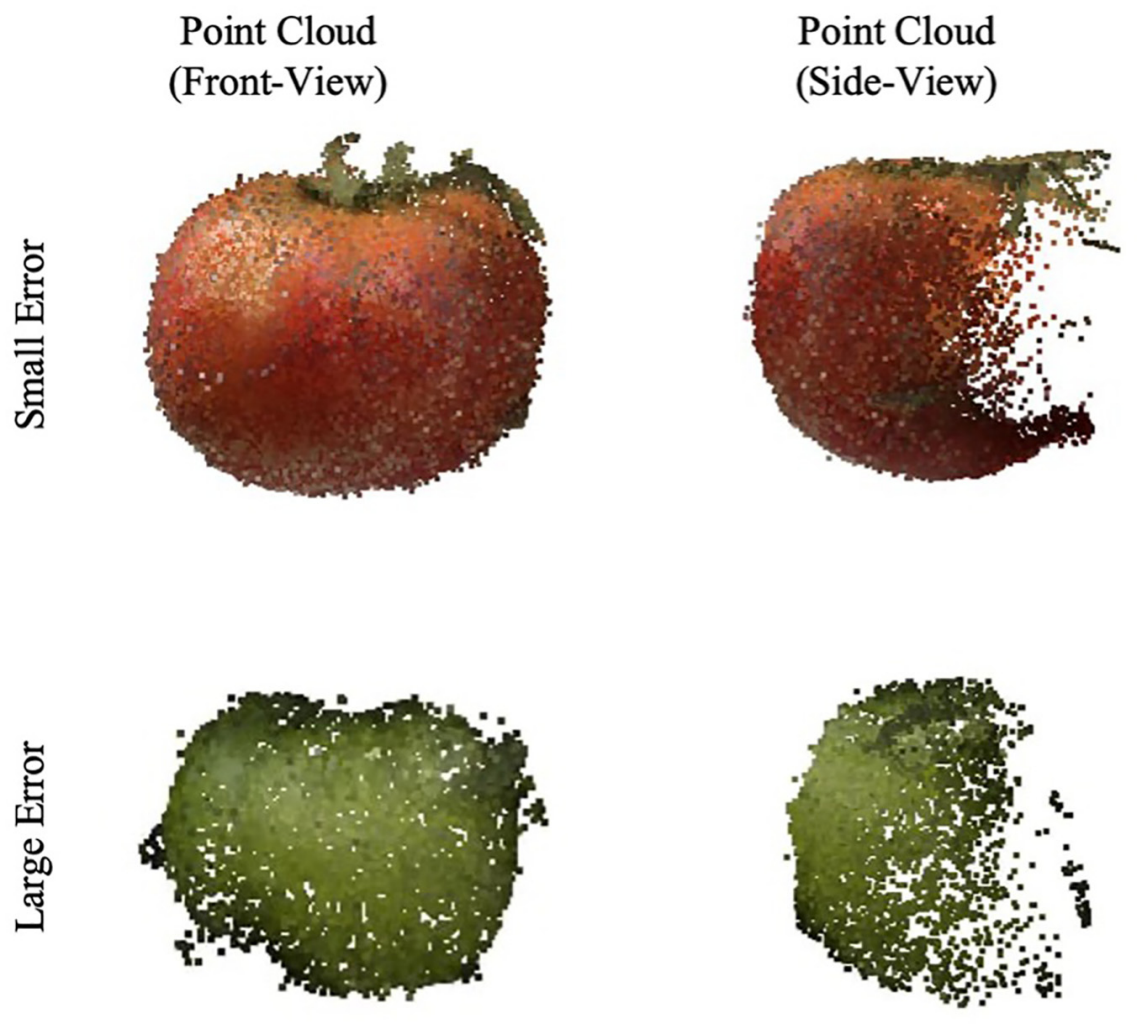
Example of tomato fruit’s point cloud. A good example with a small error is at the top, and a bad example is at the bottom.

**Figure 10** shows examples of segmented tomato fruits. Similar to the previous examples with leaves, the top two samples represent instances with minimal errors, whereas the bottom two samples show substantial discrepancies. The high errors likely resulted from the fruits being partially obscured by leaves or adjacent fruits, leading to fewer data points being captured and, consequently, errors in the fitting process.

**Figure 11:**
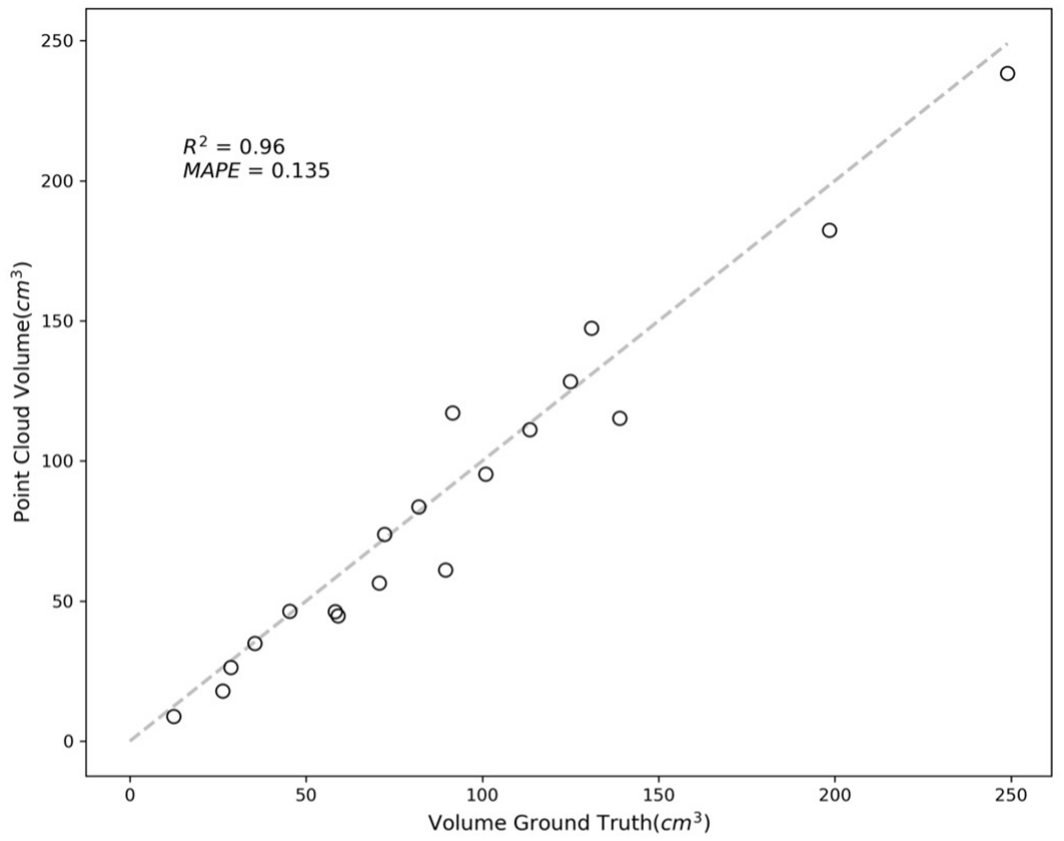
Comparison between volume from NeRF and ground truth.

In **Figure 11**, the results are quantified, showing an R-squared value of 0.96 and a MAPE of 0.135. These values indicate a high degree of accuracy in most cases, with errors primarily arising from occluded or partially hidden portions of the fruits.

The issues encountered, predominantly owing to obscured parts of the fruit, suggest a potential avenue for improvement in future studies. Addressing this challenge may involve fitting parametric geometric models to the fruits or implementing appropriate interpolation methods. Such techniques can help accurately estimate the shape and volume of partially obscured fruits, enhancing the phenotyping precision. This approach is beneficial in complex agricultural environments where occlusion by leaves or other fruits is common.

## 4 Discussion

### 4.1 Performance

The 6-axis robotic arm mounted on the SmartFarm robotic platform used in this study can capture images at 64 poses in approximately 240 seconds. Due to its general-purpose design, not specifically optimized for capturing images quickly, the image acquisition process is relatively time-consuming. However, the development of dedicated image acquisition hardware could significantly enhance both image acquisition speed. For example, implementing a system with rails capable of Z-axis movement and a camera unit with Pan-Tilt functionality could be considered. This system can perform multi-angle image acquisition in conjunction with the robot’s movement on the ground. This approach will not only reduce the time required for multi-angle image acquisition but also lower hardware costs.

### 4.2 Multi-modal point cloud

If an additional camera, such as an IR or multispectral camera, is installed parallel to the RGB camera when acquiring images, it becomes feasible to implement a multimodal point cloud. In the NeRF point cloud construction process mentioned above, the point cloud is sampled by mapping the RGB image and depth image generated as the output of NeRF. By aligning multimodal images taken from the same angle with the generated RGB image, it is possible to obtain a point cloud where the color from RGB is replaced by multimodal values. Specifically Thermal imaging using IR camera can be used to extract physiological indicators from plants (Pradawet et al., 2023). However, when these thermal data are integrated into a 3D structure, enabling the point cloud to include both morphological and physiological information, there is potential to develop more sophisticated indicators for analyzing plant stress or disease, which could lead to more accurate and representative plant physiological assessments.

### 4.3 Limitations and future work

Our current study, while demonstrating the potential of NeRF-based 3D reconstruction for tomato crop phenotyping, has several limitations that need to be addressed in future research. One limitation is that the process of extracting regions of interest from the point cloud or node graph is currently manual. This manual process introduces the potential for human error and limits scalability. To enable high-throughput phenotyping, it will be essential to incorporate additional technologies, such as AI-driven 3D segmentation (Xie et al., 2020), which could automate this process and significantly improve efficiency.

Another limitation lies in the image acquisition process, which is constrained by the robot’s fixed position, capturing images only from angles within the reach of the robotic arm. While this method has proven effective for capturing the structure of plant nodes in the upper parts of the crops, it struggles with densely vegetated lower parts where leaves and other foliage can obscure key details. The occlusion effects caused by tightly packed leaves can result in sparse point clouds and reduced accuracy in the final 3D model. The fine details of smaller leaves are particularly prone to being smoothed out or lost during the reconstruction process, further complicating accurate representation. To address these challenges, one approach could involve applying models that can efficiently process more numerous and detailed input images (Wang et al., 2022), thereby capturing finer details and reducing occlusion issues. Another potential solution is to integrate autonomous navigation technologies such as SLAM (Simultaneous Localization and Mapping). By using SLAM (Campos et al., 2021), the robot could link image sets captured from different locations, such as combining images taken from the opposite lane of the target crop, to provide a more complete view.

Additionally, the current pipeline is specifically designed for tomato crops in a greenhouse environment, with limitations in accurately measuring fruit volumes in the lower parts of the plants. Expanding the applicability of this pipeline will require more robust 3D data acquisition methods, possibly through enhanced image coverage using autonomous mobility or more sophisticated interpolation techniques, to provide comprehensive volumetric data.

Lastly, we encountered challenges related to crop movement during image capture in real greenhouse environments. Even slight movements of the crops during shooting introduced noise into the 3D reconstruction results, which compromised accuracy. To address this vulnerability, applying dynamic NeRF (Pumarola et al., 2021) that add a time axis to the radiance fields could allow the system to capture the geometry of moving objects. If integrated into 3D phenotyping, this approach could enable the system to operate robustly even in open fields where wind and crop movement are factors, offering a promising direction for future research.

## 5 Conclusion

By employing a state-of-the-art combination of NeRF technology and autonomous robotic systems, we successfully developed a pipeline capable of capturing comprehensive morphological crop data from limited viewpoints. The precision of our method was validated by the R-squared values above 0.953 and MAPE under 0.96 for length, area, and volume measurements, demonstrating its superiority over traditional growth surveys. However, our study identified challenges, such as occlusion and incomplete data capture due to foliage, indicating areas for future enhancement. Potential improvements could involve integrating parametric geometric modeling or sophisticated interpolation methods for more accurate shape and volume estimations of partially visible fruits. Overall, this research proves the viability of advanced 3D phenotyping in real-world greenhouse scenarios and paves the way for future developments in digital agriculture to optimize crop management and yield through precise morphological assessments.

## Data Availability

The raw data supporting the conclusions of this article will be made available by the authors, without undue reservation.
